# Ultra-Small Iron-Based Nanoparticles with Mild Photothermal-Enhanced Cascade Enzyme-Mimic Reactions for Tumor Therapy

**DOI:** 10.3390/ma18071649

**Published:** 2025-04-03

**Authors:** Jing Yu, Shuangshan Li, Xun Zhu, Hongyan Yu, Hao Gao, Jiarui Qi, Yao Ying, Liang Qiao, Jingwu Zheng, Juan Li, Shenglei Che

**Affiliations:** 1 Research Center of Magnetic and Electronic Materials, Zhejiang University of Technology, Hangzhou 310014, China; 2111725052@zjut.edu.cn (S.L.); zhuxun289825443@foxmail.com (X.Z.); 211122250041@zjut.edu.cn (H.Y.); 211122250092@zjut.edu.cn (H.G.); 221123250104@zjut.edu.cn (J.Q.); yying@zjut.edu.cn (Y.Y.); lqiao@zjut.edu.cn (L.Q.); zhengjw@zjut.edu.cn (J.Z.); juanli@zjut.edu.cn (J.L.); 2 College of Materials Science and Engineering, Zhejiang University of Technology, Hangzhou 310014, China

**Keywords:** ultra-small iron-based nanoparticles, chemodynamic therapy, superoxide dismutase-mimic, peroxidase-mimic, enzymatic activity enhancement

## Abstract

Chemodynamic therapy (CDT), which utilizes the catalytic reactions of nanoparticles to inhibit tumor growth, is a promising approach in cancer therapy. However, its efficacy is limited by insufficient hydrogen peroxide (H_2_O_2_) concentration in tumor microenvironments and unsatisfactory enzymatic catalytic activity. To overcome these limitations, ultra-small iron-based (USIB) nanoparticles with cascaded superoxide dismutase (SOD)-mimic and peroxidase (POD)-mimic activities have been engineered. USIB nanoparticles initiated by SOD-mimic activity to transform superoxide anions (O_2_^·−^) into H_2_O_2_, elevating H_2_O_2_ levels in the tumor microenvironment and subsequently utilizing POD-mimic activity to convert H_2_O_2_ into the more reactive ·OH, thereby achieving the destruction of tumor cells. In addition, USIB nanoparticles possess photothermal conversion capabilities, and their enzymatic activity can be significantly enhanced under mild laser irradiation. Therefore, by addressing the issues of insufficient substrate concentration and low enzymatic catalytic activity, the therapeutic efficiency of CDT has been improved. Our research integrates the cascade catalytic reactions of nanozymes with laser irradiation, effectively inhibiting tumor growth and exhibiting outstanding biosafety, demonstrating promising therapeutic potential.

## 1. Introduction

Chemodynamic therapy (CDT) is an innovative cancer treatment strategy that utilizes catalytically active nanomaterials to generate highly reactive hydroxyl radicals (·OH) by the decomposition of the abundant hydrogen peroxide (H_2_O_2_) found in the tumor microenvironment (TME), which is effective in destroying tumor cells [1,2,3,4]. However, recent research has indicated that the concentrations of H_2_O_2_ in TME varied from 1 μM to 100 μM, remaining inadequate to produce a sufficient amount of ·OH for an effective CDT [5,6,7,8]. In addition, the catalytic activity of native enzyme for decomposing H_2_O_2_ to ·OH is low, and therefore, the core of CDT is the development of mimic enzymes with improved catalytic activities using nanomaterials [9,10]. While the insufficient catalytic activity of most developed mimic enzymes still results in unsatisfactory ·OH generation [11,12]. Furthermore, the low catalytic activity of nanomaterials also results in unsatisfactory CDT outcomes. Consequently, the design and development of nanomaterial systems that address these current limitations are urgently needed.

The generation and consumption of H_2_O_2_ in tumor cells are maintained in a relatively stable state through a complex redox system, which involves various redox enzymes such as superoxide dismutase (SOD) [13,14,15], catalase (CAT) [16,17,18], peroxidase (POD) [19,20,21], and glutathione peroxidase (GPx) [22,23,24]. SOD catalyzes the conversion of superoxide anions (O_2_^·−^) [25,26,27,28], the initial product of abnormal cellular metabolism, into H_2_O_2_, thereby increasing H_2_O_2_ available for CDT catalytic reactions [29,30,31]. This H_2_O_2_ concentration improvement paves the way for nanomaterials to activate their POD-mimicking enzymatic functions by increasing the substrate, producing additional ·OH, which in turn boosts the efficacy of CDT [24,32,33]. Thus, nanomaterials that possess both SOD-mimic and POD-mimic enzymatic activities are show promise for enhancing the effectiveness of CDT. However, SOD is essentially a dismutase that reduces oxidative stress by scavenging superoxide free radicals, while POD is an oxidase that decomposes H_2_O_2_ into the more oxidizing ·OH. The radically different redox properties between SOD and POD make it difficult to combine SOD and POD within one nanoparticle, especially in highly active ultrasmall nanoparticles with a size below 5 nm [34,35,36].

Photothermal therapy (PTT) is an effective and minimally invasive tumor treatment method that offers spatiotemporal precision through its unique ability to convert light energy into heat for targeted ablation [37,38,39]. However, traditional PTT requires raising the temperature above a critical threshold (>50 °C) to effectively destroy tumors, which can inadvertently trigger inflammatory and immune responses [40,41,42,43]. To overcome these limitations, we have chosen mild laser irradiation as an external energy source to stimulate the cascade enzymatic activities of nanomaterials. This approach avoids the negative impacts associated with high photothermal effects while ensuring the effective destruction of tumor cells.

**Scheme 1:**
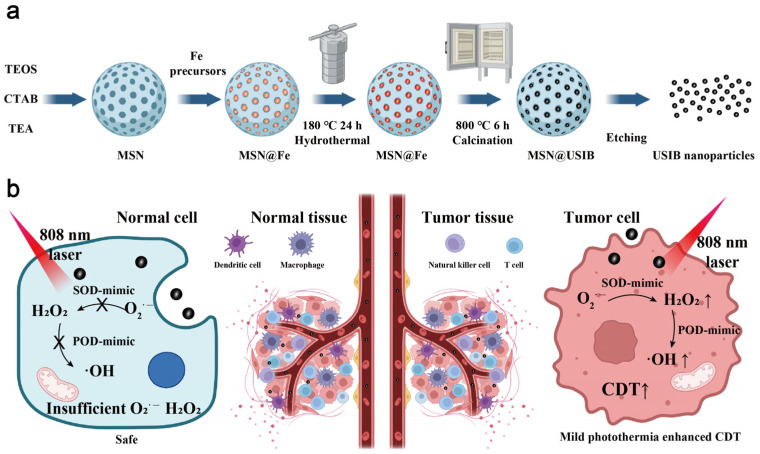
(**a**) Schematic illustration of the fabrication process of USIB nanoparticles (TEOS—tetraethyl orthosilicate; CTAB—hexadecyl trimethyl ammonium bromide; TEA—triethanolamine; MSN—mesoporous silica nanoparticle; USIB—ultra-small iron-based). (**b**) Schematic illustration of mild photothermia-enhanced SOD-POD mimic cascade enzymatic reaction, leading to improved CDT effect. This figure was created using BioRender http://app.biorender.com (accessed on 26 November 2024).

Herein, we have designed and synthesized ultra-small iron-based (USIB) nanoparticles with enhanced cascaded SOD-POD mimic activities under mild photothermal conditions (<40 °C), thereby improving the efficiency of CDT for effective tumor ablation. These nanoparticles are 4 nm in size, exhibiting a core–shell structure with a Fe_3_O_4_ core and amorphous shell. The multiple structure endows the combination of SOD and POD intrapartically. They initially improve H_2_O_2_ concentration by converting endogenous O_2_^·−^ in tumor cells into H_2_O_2_ through SOD-mimic activity. Concurrently, USIB nanoparticles exhibit POD-mimic activity to transform formed H_2_O_2_ into the highly oxidative ·OH. Interestingly, USIB nanoparticles also display photothermal conversion properties, and after irradiation with an 808 nm laser, the temperature increased, and their cascaded SOD-POD mimic activity is moderately enhanced. Notably, the cytotoxic effect on tumors is not achieved through photothermal effects, but rather through laser irradiation that enhances the enzymatic activity of the nanoparticles, facilitating the process through chemical catalytic reactions. Furthermore, we have verified the biosafety of USIB nanoparticles and their selective cytotoxic effects on tumor cells. Subsequent animal experimental results also indicated that the combined approach of CDT and laser irradiation effectively inhibited tumor growth (Scheme 1). The SOD-POD cascade system effectively resolves the insufficient substrate concentration in CDT, while laser irradiation synergistically enhances enzymatic activity, thereby improving nanozyme-mediated CDT efficacy.

## 2. Materials and Methods

### 2.1. Chemical Materials and Reagents

Tetraethyl orthosilicate (TEOS), hexadecyl trimethyl ammonium bromide (CTAB), triethanolamine (TEA), sodium hydroxide (NaOH) methylene blue (MB), pyrogallol, and trolamine were purchased from Aladdin (Shanghai, China). Ferric nitrate nonahydrate (Fe(NO_3_)_3_·9H_2_O), iron acetylacetonate (Fe(acac)_3_), 3,3′,5,5′-tetramethylbenzidine (TMB), N-acetyl cysteine (NAC) cytochrome C, xanthine, and xanthine oxidase was purchased from Sigma-Aldrich (Shanghai, China). 3,4-dihydroxybenzoic acid, hydrogen peroxide (H_2_O_2_), Dimethyl sulfoxide (DMSO), and hydrochloric acid were purchased from Sinopharm (Beijing, China). SOD assay kits were bought from Dojindo (Mashiki, Japan). The hydrogen peroxide assay kit, methylthiazolyldiphenyl-tetrazolium bromide (MTT), Calcein-AM/PI assay kit, Amplex Red assay kit, Hoechst 33258, DCFH-DA, Mito-Tracker Red CMXRos, and JC-1 were purchased from Beyotime Biotechnology, Shanghai, China.

### 2.2. Synthesis of USIB Nanoparticles

Preparation of mesoporous silica nanoparticle (MSN): A solution was prepared by dissolving cetyltrimethylammonium bromide (CTAB, 1.57 g) and triethylamine (TEA, 0.182 g) in deionized water (100 mL) under magnetic stirring at 80 °C for 1 h. Subsequently, tetraethyl orthosilicate (TEOS, 14.44 g) was added dropwise to the mixture, and the reaction was maintained at 80 °C for an additional 2 h. The resulting products were isolated via centrifugation at 12,000 rpm for 5 min and were thoroughly washed with deionized water three times to remove any residual reactants or by-products. After drying, the products were ground into powder and calcined at 600 °C for 6 h to remove the soft template CTAB.

Preparation of USIB nanoparticles: Iron (III) acetylacetonate (Fe(acac)_3_, 0.1 g) was dissolved in anhydrous ethanol (10 mL), while iron (III) nitrate nonahydrate (Fe(NO_3_)_3_·9H_2_O 0.4 g) was dissolved in deionized water (10 mL). The two solutions were subsequently combined and homogenized via ultrasonication. MSNs were then added to the mixture and stirred for 1 h. The resulting suspension was subjected to hydrothermal treatment at 180 °C for 24 h. The products were separated by centrifugation (12,000 rpm, 5 min) and washed with deionized water for three times. After drying, the products were ground into a fine powder and calcined at 800 °C for 3 h. Next, an etching solution was prepared by dissolving sodium hydroxide (NaOH, 5 g) and 3,4-dihydroxybenzoic acid (0.5 g) in deionized water (50 mL). The calcined powder was immersed in 20 mL of the etching solution and stirred for 24 h to remove the hard template MSN. The final products were collected via dialysis and subsequently processed by freeze-drying.

### 2.3. Characterization

Transmission electron microscopy (TEM, FEI Tecnai G2 F30, GFZ, Potsdam, Germany) was used to characterize the structure and morphology of USIB nanoparticles (100 µL SUIB nanoparticles solution (1 mg/mL) in n-hexane was dropped on a carbon-coated copper grid for sample preparation). X-ray diffraction (XRD) data were recorded by a PANalytical Empyrean powder diffractometer (Malvern Panalytical, Tokyo, Japan) with Cu K*α* radiation (*λ* = 0.1541 nm) (50 mg USIB nanoparticle powder was evenly spread on a cover glass for sample preparation). X-ray photoelectron spectroscopy (XPS, Thermo Scientific K-Alpha, Thermo Fisher Scientific, Waltham, MA, USA) was used to investigate the chemical composition of USIB nanoparticles (20 mg of USIB nanoparticles powder for testing). The Brunauer–Emmett–Teller (BET) curve was recorded by a Micromeritics TriStar II Plus 3.03 (Micromeritics Instrument Corporation, Norcross, GA, USA) (20 mg of USIB nanoparticles powder for testing). Fourier transform infrared (FT-IR) spectroscopy data were recorded by a Thermo Nicolet iS 10 (Thermo Fisher Scientific, Waltham, MA, USA) (20 mg of USIB nanoparticles powder for testing). Ultraviolet-visible (UV-Vis) spectroscopy data were recorded by a Persee TU1810 (Beijing, China) and SpectraMax 190 (Beijing, China). Infrared thermal imaging (ITI) and temperature data were recorded by a FLUKE Ti400 (FLUKE, Everett, WA, USA).

### 2.4. Photothermal Performance of USIB Nanoparticles

Temperature changes in USIB nanoparticle solutions (0, 40, 100, 200, 400, 800 μg mL^−1^) were recorded under laser irradiation (0.5, 1, 1.5 W cm^−2^). ITI was acquired by an infrared thermal imaging camera. A USIB nanoparticle solution (100 μg mL^−1^) was placed under laser irradiation (1 W cm^−2^) to evaluate the photothermal conversion capability.

### 2.5. Superoxide Dismutase Mimic Activity of USIB Nanoparticles

The xanthine/xanthine oxidase system was used to calculate the specific activity of USIB. O_2_^·−^ inhibition of USIB nanoparticles was measured by a SOD detection kit, according to the manufacturer’s instructions.

The SOD-mimic activity was also measured by the pyrogallol oxidation method. A measure of 2.95 mL USIB nanoparticles solution (100, 200, 300, 400, 500 μg mL^−1^, in Tris-HCl buffer 50 mmol L^−1^) and 50 μL pyrogallol solution (60 mmol L^−1^) were added to quartz cuvettes and mixed completely. The final volume was 3 mL. A UV-Vis spectrophotometer (Persee TU1810) was used to read the absorbance at 325 nm every 30 s until 300 s. O_2_^·−^ inhibition rate = (A_control_ − A_USIB_)/(A_control_) × 100%.

H_2_O_2_ generation detection: The generation of H_2_O_2_ was measured by the hydrogen peroxide assay kit, according to the manufacturer’s instructions. First, the H_2_O_2_ solution (0, 0.5, 1, 1.5, 2, 2.5 mM) absorbance was measured at 560 nm to establish a standard curve using a plate reader. Next, 50 μL USIB solution (100, 200, 300, 400, 500 μg mL^−1^) mixed with xanthine/xanthine oxidase was placed in a 96-well plate. Then, 100 μL of H_2_O_2_ detection reagent was added to each well, mixed by shaking, and left to stand at room temperature for 30 min. Finally, the plate reader (SpectraMax190) was used to measure the absorbance at 560 nm and the H_2_O_2_ production was calculated based on the standard curve.

### 2.6. Peroxidase-Mimic Activity of USIB Nanoparticles

TMB was used to assess the peroxide-mimic activity of USIB nanoparticles. USIB nanoparticle solution (100 μg mL^−1^, 1 mL), TMB in DMSO solution (10 mg mL^−1^, 40 μL), and H_2_O_2_ (50 mmol L^−1^) were added into acetate buffer (pH 4.5, pH 5.4); the final volume was 4 mL. The absorption spectrum was recorded by UV-Vis spectrophotometer.

Steady-state kinetic assay: According to the national standard of China (GB/T 37966-2019) [44], with varying concentrations of H_2_O_2_ as the substrate, the changes in absorbance at 652 nm were measured. USIB nanoparticle solution (100 μg mL^−1^, 1 mL), TMB in DMSO solution (10 mg mL^−1^, 40 μL), and H_2_O_2_ solutions with different concentration gradients (10, 25, 50, 75, 100, 150, 200, 300, 400, 500, 600, 700, 800, 900, 1000 mmol L^−1^) were added into the acetate buffer (pH 4.5); the final volume was 4 mL. The Michaelis constant (*K*_m_) and maximum reaction velocity (*v*_max_) are critical parameters in steady-state kinetics. *K*_m_ and *v*_max_ were calculated using the Michaelis–Menten Equation, as follows:

$$v=(v_{max}\times \left[S\right])/(K_{m}+[S])$$

where *v* is the initial reaction velocity and *v*_max_ is the maximal reaction rate that is observed at saturating substrate concentrations. [*S*] is the concentration of the substrate and *K*_m_ is the Michaelis constant. *K*_m_ reflects the affinity of the nanozyme for its substrate and is defined as the substrate concentration at half the maximum rate.

OH generation detection: MB was used to assess the ·OH generation of USIB nanoparticles. USIB nanoparticle solution (100 μg mL^−1^, 1 mL), MB (5 mg mL^−1^), and H_2_O_2_ (10 mmol L^−1^) were placed in PBS buffer (pH 5.4). Absorbance at 665 nm was measured using a UV-Vis spectrophotometer.

### 2.7. VSM and MRI Experiments

Saturation magnetization measurement: The magnetic properties of the USIB nanoparticles were analyzed using a vibrating sample magnetometer (VSM, LakeShore-8604, Lake Shore Cryotronics, Inc., Westerville, OH, USA). Briefly, 10 mg of USIB nanoparticles were loaded into a quartz sample holder. Magnetization versus applied field (M-H) curves were recorded at room temperature under a sweeping magnetic field ranging from −20 kOe to 20 kOe.

MRI performance testing: The longitudinal relaxivity (*r*_1_) of the USIB nanoparticles was evaluated using a 3.0 T clinical MRI scanner (Philips, Amsterdam, The Netherlands). USIB nanoparticles were dispersed in deionized water at iron concentrations from 0.0175 mM to 0.35 mM (∆_concentration_ = 0.0175 mM). Each concentration (1 mL) was transferred to a 1.5 mL centrifuge tube for scanning. The parameters of a T_1_-weighted sequence were set as follows: echo time (TE) = 11 ms; repetition time (TR) = 500 ms; field of view (FOV) = 200 mm; slice thickness = 2 mm; flip angle = 70°. The parameters of a T_2_-weighted sequence were set as follows: echo time (TE) = 90 ms; repetition time (TR) = 2.5 ms; field of view (FOV) = 200 mm; slice thickness = 2 mm; flip angle = 90°.

### 2.8. Cell Culture

Mouse breast cancer (4T1) cells and mouse fibroblast (L929) cells were received from Zhejiang Provincial People’s Hospital, Hangzhou, China. The 4T1 and L929 cells were seeded in RPMI 1640 medium and Dulbecco’s modified eagle medium containing 10% fetal bovine serum and 1% penicillin–streptomycin, respectively. The cell lines were incubated at 37 °C under an atmosphere of 5% CO_2_.

### 2.9. Cellular Uptake Assay

FITC labeling of USIB nanoparticles: A measure of 50 mg of USIB nanoparticles were dispersed in 5 mL of carbonate buffer solution (pH 9.7) under sonication. Then, 2 mL FITC solution (1 mg/mL in DMSO) was introduced dropwise into the nanoparticle suspension while maintaining ultrasonic agitation. The reaction mixture was protected from light and continuously shaken for 12 h at 25 °C to facilitate covalent conjugation between FITC and USIB nanoparticles. Subsequent purification was achieved through three cycles of centrifugal washing (8000 rpm, 15 min) using absolute ethanol and drying to obtain the FITC-labeled USIB nanoparticles.

4T1 cells were seeded in a 12-well plate containing 1 × 10^5^ cells each well and cultured overnight. Then, the medium was replaced with 1 mL of fresh culture medium containing FITC-labeled USIB nanoparticles (100 μg mL^−1^) and co-cultured for various time intervals (0, 1, 2, and 4 h). To visualize the cell nuclei, 500 μL Hoechst 33258 staining (10 μg mL^−1^) was applied for 30 min. The fluorescence images were recorded employing an inverted fluorescence microscope. FITC: *λ*_ex_ = 490 nm; *λ*_em_ = 520 nm. Hoechst 33258: *λ*_ex_ = 346 nm; *λ*_em_ = 460 nm.

### 2.10. MTT Assay

4T1 cells and L929 cells were seeded into a 96-well plate at a density of 5 × 10^3^ cells per well and then treated with varying concentrations of USIB nanoparticles (0, 8, 16, 24, 32, 40 μg mL^−1^) and exposed to 808 nm laser irradiation at a power density of 1 W cm^−2^ for 5 min. Following a 24 h incubation at 37 °C, the cells were washed with PBS, and 100 µL of MTT solution (1 mg mL^−1^) was added to each well. Following a 4 h incubation, the absorbance at 490 nm was measured using a plate reader to determine the cell survival rate.

### 2.11. Cell Imaging

Intracellular H_2_O_2_ Staining assay: 4T1 cells were pre-seeded in 6-well plates at a density of 2 × 10^5^ cells per well and incubated for 24 h. USIB nanoparticles (40 μg mL^−1^) in DMEM medium (without 10% FBS) incubated with cells for 0, 0.5, 1, 2, 4, and 6 h. Then, 1 mL Amplex Red (100 μg mL^−1^) was used to stain the intracellular H_2_O_2_ level for 30 min. The fluorescence images were recorded employing an inverted fluorescence microscope (EVOS M5000, Thermo Fisher Scientific). Amplex Red: *λ*_ex_ = 571 nm; *λ*_em_ = 585 nm.

Intracellular ROS Staining assay: 4T1 cells were pre-seeded in 6-well plates at a density of 2 × 10^5^ cells per well and incubated for 24 h. USIB nanoparticles (40 μg mL^−1^) in DMEM medium (without 10% FBS) were incubated with cells for 4 h. Then, an 808 nm laser (1 W cm^−2^) was employed for 5 min and incubated for 2 h. Following this, 1 mL DCFH-DA (10 μmol L^−1^) was used to stain the intracellular ROS level for 30 min. The fluorescence images were recorded employing an inverted fluorescence microscope (EVOS M5000). DCFH-DA: *λ*_ex_ = 488 nm; *λ*_em_ = 525 nm.

Live/dead cell staining assay: To visualize the viable cells and dead cells after 4T1 cells accepted the above protocol treatments, 1 mL Calcein-AM/Propidium iodide (PI) staining reagents were applied to stain the live cells as green fluorescence and dead cells as red fluorescence for 30 min. The fluorescence images were recorded employing an inverted fluorescence microscope (EVOS M5000). Calcein-AM: *λ*_ex_ = 494 nm; *λ*_em_ = 517 nm. PI: *λ*_ex_ = 535 nm; *λ*_em_ = 617 nm.

Mitochondrial membrane potential staining assay: 4T1 cells underwent the above protocol treatments. A measure of 1 mL Hoechst 33258 staining (10 μg mL^−1^) was applied to stain the nuclei for 30 min. A measure of 1 mL Mito-Tracker Red CMXRos (100 nmol L^−1^) was applied to stain the mitochondria for 30 min. The fluorescence images were recorded employing an inverted fluorescence microscope (EVOS M5000). Calcein-AM: *λ*_ex_ = 494 nm; *λ*_em_ = 517 nm. PI: *λ*_ex_ = 535 nm; *λ*_em_ = 617 nm. Hoechst 33258: *λ*_ex_ = 346 nm; *λ*_em_ = 460 nm. Mito-Tracker Red CMXRos: *λ*_ex_ = 579 nm; *λ*_em_ = 599 nm.

Mitochondrial integrity staining assay: 4T1 cells underwent the above protocol treatments. A measure of 1 mL JC-1 (10 μg mL^−1^) was applied to stain the mitochondria for 30 min according to the manufacturer’s protocol. The fluorescence images were recorded employing an inverted fluorescence microscope (EVOS M5000). JC-1 monomers: *λ*_ex_ = 514 nm; *λ*_em_ = 529 nm. JC-1 aggregates: *λ*_ex_ = 585 nm; *λ*_em_ = 590 nm.

### 2.12. Flow Cytometry Analysis

4T1 cells were plated in a 6-well plate at a density of 2 × 10^5^ cells per well and cultured for 24 h. 4T1 cells accepted the above protocol treatments. To detect apoptosis, cells were incubated with Annexin V-FITC and propidium iodide (PI) for 30 min. The cells were subsequently analyzed using flow cytometry to quantify the apoptotic response.

### 2.13. Biosafety Assay

The experimental operations in vivo were approved by the Institutional Animal Ethics Committee (IAEC) of Zhejiang University of Technology, Hangzhou, China (Animal ethics application number: MGS20230730114). Eighteen BALB/c mice were randomly divided into three groups, with six mice per group: (1) Control, (2) USIB (5 mg kg^−1^), (3) USIB (10 mg kg^−1^). PBS and different concentrations (5, 10 mg/kg) of USIB nanoparticle solution were administered to the mice via tail vein injection at a volume of 100 μL per injection. Injections were administered every 3 days for a total of 5 doses over a 16-day experimental period.

Hemolysis assay: USIB nanoparticle solutions at different concentrations (0, 50, 100, 150, 200, 300 μg/mL) were prepared using sterile PBS and added to 1.5 mL centrifuge tubes. Deionized (DI) water and PBS served as control groups. Fresh mouse erythrocytes were mixed with each solution (1:9 *v*/*v*) in 1.5 mL centrifuge tubes and incubated at 37 °C for 3 h, followed by centrifugation at 4000 rpm for 5 min. Photographs of the supernatants were taken to qualitatively assess hemolysis. Supernatants (100 μL per well) were transferred to a 96-well plate (*n* = 3). Absorbance at 541 nm was measured using a plate reader (SpectraMax 190). Hemolysis rates (%) were calculated using the following formula: (A_sample_ − A_PBS_)/(A_DIwater_ − A_PBS_) × 100.

Biochemical and hematological marker analysis: At the endpoint (Day 16), blood samples were collected via retro-orbital bleeding. Biochemical and hematological markers were analyzed using an automated hematology analyzer.

Histopathological analysis: Mice were euthanized by cervical dislocation. Major organs (heart, liver, spleen, lungs, kidneys) were harvested, fixed in 4% paraformaldehyde for 24 h, and embedded in paraffin. Tissue sections were stained with hematoxylin and eosin (H&E) staining.

### 2.14. In Vivo MRI

MRI experiments on 4T1 tumor-bearing mice were performed using a 9-T MRI scanner (Timemedical, Hong Kong, 9 T/110). Mice were anesthetized via inhalation of a 1.5% isoflurane-oxygen mixture and maintained under anesthesia throughout the procedure. Then, 100 μL USIB nanoparticle solution (10 mg kg^−1^) was intravenously administered via the tail vein. To obtain the images, scanning was performed at different time points (0, 1, 2, 4, 6, 8, 12, and 24 h) using a T₁-weighted sequence with the following parameters: repetition time (TR) = 350 ms, echo time (TE) = 8 ms, and slice thickness = 1.0 mm.

### 2.15. In Vivo Therapeutic Evaluation of USIB Nanoparticles

Twenty-five 4T1 tumor-bearing mice were randomly divided into four groups (*n* = 5 per group) as follows: (1) Control (PBS), (2) Laser, (3) USIB nanoparticles, (4) USIB nanoparticles + Laser. A measure of 100 μL USIB nanoparticle solution (10 mg kg^−1^) was administered to the mice via tail vein injection. After 6 h post-injection, tumor tissues were exposed to an 808 nm laser at a power density of 1 W cm^−2^ for 5 min. During the heating process, a thermal imaging camera was employed to record temperature changes every 1 min and take thermal images of the mice. The body weights and tumor volume of mice were recorded every two days. Tumor volume (mm^3^) was calculated using the formula *V* = *lw*^2^/2, where *w* and *l* are the width and length of the tumor, respectively. After 16 days of the treatment, all mice were sacrificed for H&E, Ki67, and TUNEL staining analysis.

## 3. Results and Discussion

### 3.1. Synthesis and Characterizations

**Figure 1:**
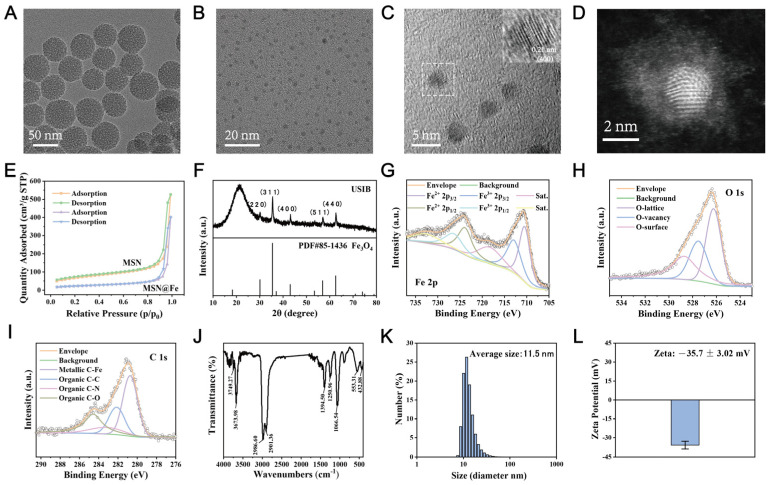
Characterization of USIB nanoparticles. (**A**) TEM image of MSN. (**B**) TEM image of USIB nanoparticles. Insert: high magnification. (**C**) High-resolution TEM image of USIB nanoparticles. (**D**) AC-HAADF-STEM image of USIB nanoparticles. (**E**) Nitrogen adsorption–desorption isotherms of MSN and MSN-USIB nanoparticles. (**F**) XRD pattern of USIB nanoparticles. (**G**) High-resolution Fe 2p XPS spectrum of USIB nanoparticles. (**H**) High-resolution O 1s XPS spectrum of USIB nanoparticles. (**I**) High-resolution C 1s XPS spectrum of USIB nanoparticles. (**J**) FTIR spectrum of USIB nanoparticles. (**K**) Hydrodynamic size of USIB nanoparticles. (**L**) Zeta potential of USIB nanoparticles.

USIB nanoparticles were synthesized using the hard template assistant method combined with hydrothermal treatment and high-temperature calcination. Initially, hard template mesoporous silica nanoparticle (MSN) was synthesized via a sol–gel method. The transmission electron microscopy (TEM) image reveals that MSN exhibits a uniform spherical morphology with an average diameter of approximately 50 nm (Figure 1A). More importantly, it was clearly observed that MSN possesses a mesoporous structure rather than a solid structure, which can be used as a template for the synthesis of ultra-small nanoparticles. The Brunauer–Emmett–Teller (BET) specific surface area, pore volume, and pore size of MSN were 232.78 m^2^/g, 0.31 cm^3^/g, 5.39 nm, respectively (Figure 1E and Figure S1A,B). Subsequently, iron precursors in the form of iron acetylacetonate and ferric nitrate nonahydrate were introduced and adsorbed within the mesoporous channels of MSN. After being calcined at 800 °C for 3 h in N_2_ atmosphere, MSN@Fe was synthesized through the carbonization of the iron precursor. The BET surface area and pore volume of MSN@Fe decreased to 76.84 m^2^/g and 0.12 cm^3^/g, respectively (Figure 1E and Figure S2A,B). The TEM images of MSN@Fe reveal distinct framework reconstruction accompanied by surface-deposited ultra-small nanoparticles, suggesting in situ iron oxide nucleation during the phase transition process (Figure S2C). Then, the USIB nanoparticles were obtained by removing the hard template MSN in alkaline solution.

As shown by TEM, the USIB nanoparticles exhibited a near-spherical shape with a diameter of approximately 4 nm (Figure 1B). The crystal structure was further characterized by high-resolution transmission electron microscopy (HRTEM). The image revealed that the lattice spacing of USIB nanoparticles is 0.21 nm, which correlated with the (400) lattice plane of face-centered cubic structure of Fe_3_O_4_ (Figure 1C). The fine structure of USIB nanoparticles was identified by aberration-corrected atomic-resolution high-angle annular dark-field scanning transmission electron microscopy (AC-HAADF-STEM, JEOL Ltd., Tokyo, Japan). As shown in Figure 1D, the nanoparticles exhibit a structure similar to a core–shell configuration, with a high-density bright core enveloped by an outer layer of low-contrast amorphous shell. The X-ray powder diffraction (XRD) pattern revealed five dominant diffraction peaks, which matched well with the face-centered cubic Fe_3_O_4_ (Figure 1F). Combined with HRTEM and AC-HAADF-STEM analyses, these results indicate that the USIB nanoparticles feature an Fe_3_O_4_ phase crystalline core, while the outer carbon layer exhibits no significant crystallinity and generates no distinct characteristic diffraction signals.

X-ray photoelectron spectroscopy (XPS) was employed to analyze the surface element composition and valence state of USIB nanoparticles. The results showed that USIB nanoparticles exist in peaks of Fe 2p, O 1s, C 1s, and N 1s, respectively (Figure S3A). The Fe 2p peak displayed two distinct peaks, which corresponded to Fe 2p_3/2_ (710.6 eV) and Fe 2p_1/2_ (724.1 eV). By fitting the Fe 2p XPS spectrum, the presence of Fe^2+^ and Fe^3+^ oxidation states were clearly determined (Figure 1G). Furthermore, the chemical coordination environments of the main constituent elements were analyzed (O 1s: Figure 1H, C 1s: Figure 1I, N 1s: Figure S3B). The high-resolution XPS spectra of O 1s revealed that oxygen species primarily exist in three distinct forms: lattice oxygen (O-lattice), oxygen vacancies (O-vacancy), and surface-adsorbed oxygen species (O-surface). Notably, the oxygen vacancies were found to account for a measurable proportion of the oxygen composition, which may exert non-negligible influences on the enzymatic activity through defect-mediated mechanisms [17,45,46]. Furthermore, the deconvoluted C 1s spectrum demonstrated the primary bonding configurations of carbon in the USIB nanoparticles are C-Fe, C-C, C-O and C-N bonds, suggesting the existence of carbon in USIB nanoparticles.

A Fourier transform infrared spectrometer (FTIR) was employed to analyze the surface functional groups of USIB nanoparticles. The presence of signals including O-H stretching vibration absorption (3673.98 cm^−1^), saturated C-H stretching vibration absorption (2986.60 cm^−1^, 2901.36 cm^−1^), C-H bending vibration absorption (1394.50 cm^−1^), and C-O stretching vibration absorption (1250.96 cm^−1^, 1066.54 cm^−1^) are observed, which are basically consistent with the XPS peak deconvolution results (Figure 1J).

In summary, the USIB nanoparticles exhibit a core–shell structure, with an Fe_3_O_4_ core and an outer carbon layer formed through the carbonization of organic compounds. Owing to the small size of the nanoparticles and the presence of numerous crystallographic defects, the USIB nanoparticles are anticipated to exhibit enhanced enzymatic activity and photothermal conversion efficiency.

The size distribution of USIB nanoparticles was measured using dynamic light scattering (DLS), revealing an average hydrodynamic diameter of 11.5 nm (Figure 1K). Zeta potential analysis showed a value of −35.7 ± 3.02 mV, indicating a negative charge on the nanoparticle surface due to the deprotonation of surface -COOH groups (Figure 1L). This negative charge facilitates electrostatic repulsion, effectively preventing aggregation of the nanoparticles. Additionally, size measurements taken over a period of seven days demonstrated the long-term stability of the nanoparticles (Figure S4).

### 3.2. Photothermal Performance of USIB Nanoparticles

**Figure 2:**
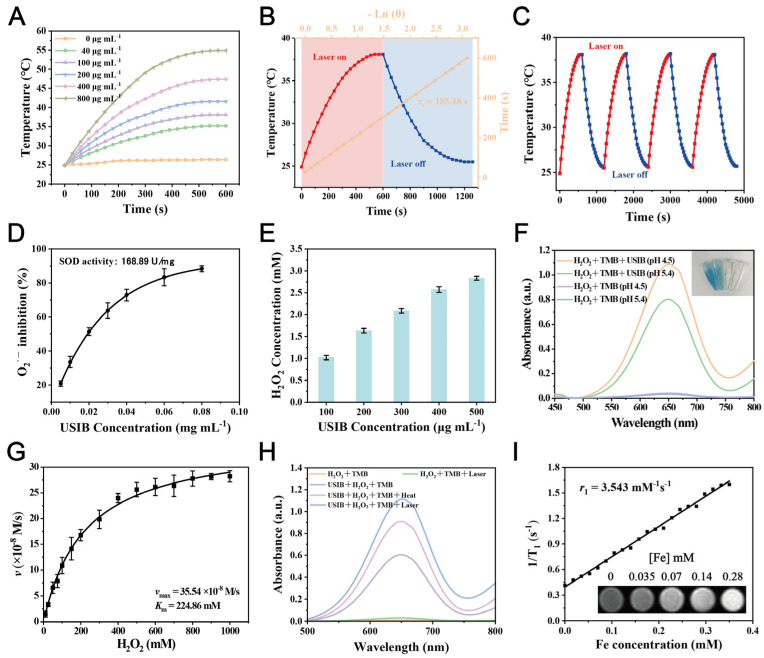
Photothermal properties and enzyme-mimic activities of USIB nanoparticles. (**A**) Photothermal conversion ability of USIB nanoparticles under 808 nm laser irradiation (1 W cm^−2^) with different concentrations (40, 100, 200, 400, 800 μg mL^−1^). (**B**) Calculation of the photothermal conversion efficiency. Red: heating curve. Blue: cooling curve. Orange: linear time (τ_s_) data obtained from the cooling period. (**C**) Recycling-heating profiles of USIB nanoparticles for four laser on/off cycles. (**D**) SOD-mimicking activity of USIB nanoparticles by measuring the inhibition rate of O_2_^·−^ (*n* = 3). (**E**) H_2_O_2_ generation profiles after treatment with USIB nanoparticles (*n* = 3). (**F**) POD-mimic activity of USIB nanoparticles and corresponding photographs (pH 4.5, pH 5.4). (**G**) Michaelis−Menten curves of UISB nanoparticles for POD-mimic activity with H_2_O_2_ as the substrate (*n* = 3). (**H**) UV-Vis absorption spectra of TMB oxidation under different treatment conditions. USIB nanoparticle concentration: 100 μg mL^−1^. Laser power density: 1 W cm^−2^. Irradiation time: 5 min. Heat temperature: 40 °C. (**I**) Plot of 1/T_1_ over Fe concentration of USIB nanoparticles and corresponding T_1_-weighted magnetic resonance images. Data presented as mean ± S.D.

The distinctive core–shell architecture of USIB nanoparticles, featuring an outer carbon layer, endows them with certain photothermal conversion capabilities. The UV-Vis-near-infrared (NIR) spectra of USIB nanoparticles are presented in Figure S5. While no distinct absorption peak was observed at 808 nm, the concentration-dependent absorption behavior confirms the broadband near-infrared light harvesting capability of USIB nanoparticles. Subsequently, we evaluated the photothermal performance of USIB nanoparticles using an 808 nm laser, which is widely used in clinical applications. Temperature elevation curves were obtained by irradiating solutions of different concentrations (0, 40, 100, 200, 400, 800 μg mL^−1^) with an NIR 808 nm laser (1 W cm^−2^) for 600 s. It was observed that solutions of varying concentrations could achieve distinct final temperatures (25.4 °C, 34.6 °C, 37.8 °C, 40.5 °C, 46.1 °C, 56.6 °C) (Figure 2A and Figure S7A). Meanwhile, the photothermal effects of USIB nanoparticles under different laser powder densities were also investigated, showing powder dependence behavior (Figures S6 and S7B). The photothermal conversion capability was assessed using a USIB aqueous solution at a concentration of 100 μg mL^−1^ under the irradiation power of 1 W cm^−2^, and the calculated photothermal conversion efficiency was 29.25% (Figure 2B). Furthermore, the photothermal curves revealed negligible variation even after four lasers on/off cycles, indicating excellent photothermal stability (Figure 2C). In conclusion, these USIB nanoparticles have an applicable photothermal conversion effect, and showed potential for use in photothermal-related therapy.

### 3.3. Enzyme-Mimic Property of USIB Nanoparticles

Considering the ultra-small size and the presence of crystal defects, we investigated the multi-enzymatic activity of USIB nanoparticles. The SOD-mimic activity of USIB nanoparticles was initially investigated by measuring the O_2_^·−^ scavenging ability produced by xanthine-xanthine oxidase systems. As shown in Figure 2D, it showed a high specific activity of 168.89 U/mg, which is crucial for the H_2_O_2_ supplement for better CDT. The SOD-mimic activity at various concentrations of nanozyme was further measured using the pyrogallol assay (Figure S8). The production of H_2_O_2_ by SOD-mimic enzyme activity of USIB nanoparticles was further affirmed by observing the H_2_O_2_ concentration at various nanozyme concentrations (Figure 2E and Figure S9).

The structure of the USIB nanoparticles, with an Fe_3_O_4_ core, endows them with excellent POD-mimic enzyme activity. Therefore, the POD-mimic activity of the nanozyme was evaluated through the 3,3′,5,5′-tetramethylbenzidine (TMB) colorimetric reaction. The POD-mimic enzyme activity of USIB nanoparticles was evident in the oxidation of TMB under acidic conditions (pH 5.4 and 4.5) (Figure 2F). To quantify the POD-mimic activity of USIB nanoparticles, steady-state kinetic analyses were performed under various H_2_O_2_ concentrations, and the resulting curve matched the typical Michaelis–Menten kinetics (Figure 2G). Interestingly, it is found that when the USIB nanoparticles (100 μg mL^−1^) were irradiated with an 808 nm laser (1 W cm^−2^) for 5 min (the solution temperature raised to 37.8 °C), the POD-mimic enzyme activity was enhanced (Figure 2H), which can partly be ascribed to the photothermal effect of USIB nanoparticles, while the temperature increase is able to accelerate the catalytic rate for some nanozymes [47,48]. Consequently, the POD-mimic enzyme activity was increased by raising the temperature to 40 °C, but the activity is still lower than that of laser irradiation (Figure 2H), indicating that the enhancement of POD activity by USIB nanoparticles under laser irradiation is not solely achieved through thermal effects. Some additional internal interactions within nanoparticles, such as internal electron transfer, also affect the generation of ·OH [49,50,51]. Subsequently, the degradation of methylene blue (MB) was achieved through the POD-mimic enzymatic reaction product ·OH, which also confirmed the POD-mimic activity (Figure S10).

### 3.4. Magnetic Properties and Magnetic Resonance Image (MRI) of USIB Nanoparticles

The magnetic properties of nanoparticles are closely related to their size. The magnetic properties of USIB nanoparticles were measured using a vibrating sample magnetometer (VSM) with a magnetic field of up to 20 kOe at room temperature. The field-dependent magnetization (M-H) curve reveals that USIB nanoparticles exhibit typical superparamagnetic behavior with a saturation magnetization (M_s_) of 58 emu/g (Figure S11A). Research reports that smaller nanoparticles exhibit a stronger spin-canting effect, which results from the incomplete alignment of spins in surface atoms, especially for nanoparticles smaller than 5 nm, making them potential candidates for T_1_ MRI contrast agents [52,53,54]. Encouraged by the ultrasmall size of USIB nanoparticles, we measured their relaxation times using a magnetic resonance scanner. T_1_-weighted MR images of USIB nanoparticles at different concentrations are shown, with higher concentrations corresponding to brighter images. The *r*_1_ and *r*_2_ relaxivities of the USIB nanoparticles are 3.543 mM^−1^ s^−1^ and 5.704 mM^−1^ s^−1^, respectively (Figure 2I and Figure S11B). The USIB nanoparticles have a small *r*_2_/*r*_1_ ratio of 1.61, indicating that USIB nanoparticles can serve as efficient T_1_ contrast agents.

### 3.5. Laser-Enhanced Cytotoxicity of USIB Nanoparticles In Vitro

**Figure 3:**
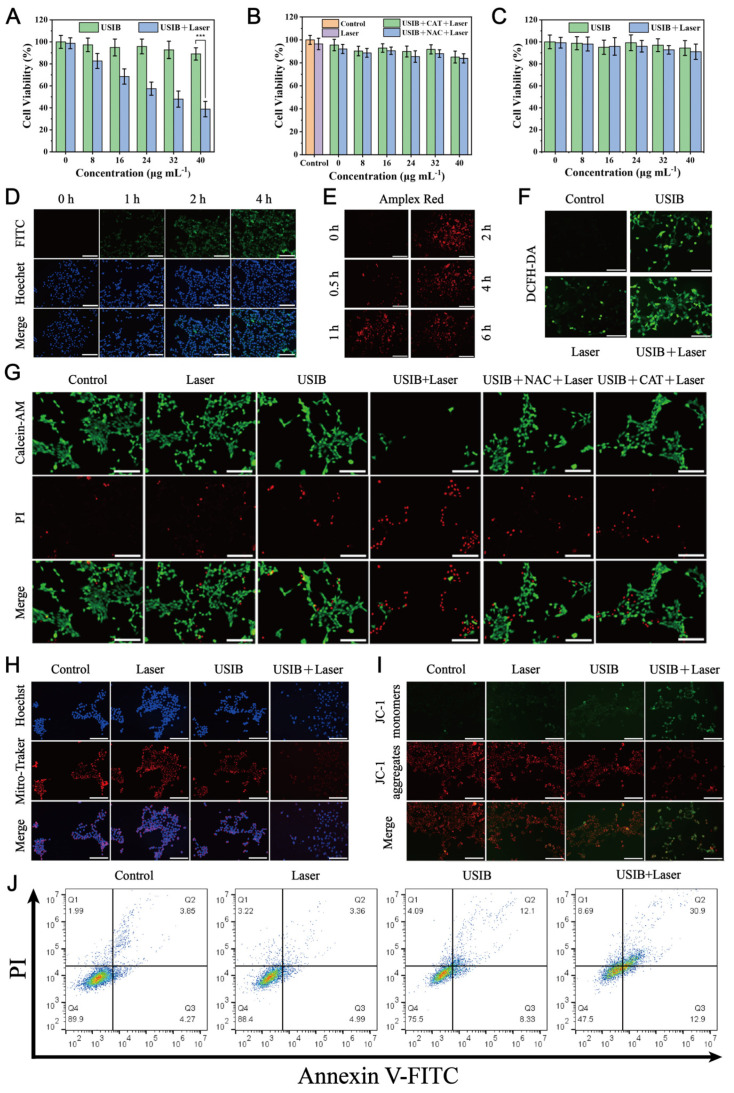
Cytotoxic effects of USIB nanoparticles in vitro. (**A**) MTT assay of 4T1 cells incubated with USIB nanoparticles, (*n* = 5). (**B**) MTT assay of 4T1 cells incubated with USIB nanoparticles, CAT (H_2_O_2_ scavenger) or NAC (OH scavenger), (*n* = 5). (**C**) MTT assay of L929 cells incubated with USIB nanoparticles, (*n* = 5). (**D**) Fluorescence images of 4T1 cells incubated with USIB nanoparticles. (**E**) Fluorescence images of intracellular H_2_O_2_ level stained with Amplex Red. (**F**) Fluorescence images of intracellular ROS level stained with DCFH-DA. (**G**) Fluorescence images of 4T1 cells stained with calcein-AM (green signal, lived cells) and PI (red signal dead cells). (**H**) Fluorescence images of 4T1 cells stained with Mito-Tracker Red CMXRos. (**I**) Fluorescence images of 4T1 cells stained with JC-1 (green signal indicating mitochondrial dysfunction). (**J**) Flow cytometry analysis of the apoptosis of 4T1 cells incubated with USIB nanoparticles. Data presented as mean ± S.D. Statistical significance was calculated by one-way ANOVA with Tukey’s multiple comparisons test, *** *p* < 0.001. All laser intensity applied was 1 W cm^−2^, and the irradiation time is 5 min. Nanoparticle concentration for Figure 3D–I was 40 μg mL^−1^. Scale bar: 150 µm.

Inspired by the excellent cascade SOD- and POD-mimic enzymic performance, as well as the NIR-enhanced enzymic activity, we further explored the enzymatic activity of USIB nanoparticles in vitro. As shown in Figure 3A, USIB nanoparticles alone had limited effects towards the growth of mouse breast cancer cells (4T1) cells, despite the fact that they possessed a cascade SOD-mimic and POD-mimic enzymic activity. At a 40 μg mL^−1^ concentration of USIB nanoparticles, cell viability remains around 90%. Interestingly, after irradiation with an 808 nm laser (1 W cm^−2^, 5 min), the cell killing effect of USIB nanoparticles was much improved, with the cell viability decreased to 40% at the concentration of 40 μg mL^−1^, which was ascribed to the photothermal effect of the nanoparticles.

Since photothermia itself is regarded as a novel tumor therapy, it is necessary to investigate whether heat effect or the increased ·OH generation improved the tumor cells’ growth inhibition. Therefore, a variety of reactive oxygen species (ROS) scavengers, including catalase (CAT, H_2_O_2_ scavenger) and N-acetylcysteine (NAC, ·OH scavenger), were introduced to co-incubation with cells. It is astonishing that the addition of CAT or NAC can totally recover the cell viability, even under NIR irradiation (Figure 3B), suggesting that the improved ·OH generation was the main reason for the better cell killing of USIB nanoparticles rather than the effect of heat. Since normal cells have less sensitivity to ROS, the improved cytotoxicity towards normal cells was invalid. The cell viability of mouse fibroblast cells (L929) incubated with USIB nanoparticles at 40 μg mL^−1^ remained higher than 90%, even the addition of NIR had almost no influence (Figure 3C), suggesting the NIR-enhanced cascade enzymatic reaction was safe for tumor therapy in vitro.

The biological mechanism for the NIR-enhanced enzyme activity was then investigated. Cellular uptake is the premise for the cytotoxicity of nanomaterials; the cellular internalization behavior of USIB nanoparticles was evaluated using FITC labeling. As shown in Figure 3D, green fluorescence signal intensity gradually increases, indicating the uptake of USIB nanoparticles by 4T1 cells. The intracellular H_2_O_2_ concentration was assessed using Amplex Red, which exhibited an initial increase within 4 h followed by a subsequent decrease in red fluorescence (Figure 3E). This phenomenon is attributed to the fact that USIB nanoparticles exhibit dual enzymatic activities, functioning as both SOD and POD mimics. This bifunctional catalytic behavior establishes a self-sustaining redox cycle; the SOD-mimic activity catalyzes the disproportionation of O_2_^·−^ into H_2_O_2_, while the subsequently generated H_2_O_2_ serves as the substrate for the POD-mimic activity, which further decomposes it into ·OH. To further confirm the mechanism of tumor cell death induced by USIB nanoparticles, intracellular ·OH levels were investigated using fluorescence imaging with DCFH-DA as the probe. The significantly enhanced green fluorescence intensity after treatment with USIB nanoparticles and laser irradiation confirmed the elevated ·OH levels (Figure 3F). The combination of USIB with laser irradiation was evidenced by using calcein-AM/propidium iodide (PI) staining to differentiate live and dead cells, which showed a dim green and obvious red fluorescence emitted from the USIB + laser group (Figure 3G). The addition of ROS scavengers (NAC or CAT) reversed cell death, further proving that improved ROS generation was the essence for the cell killing.

Since mitochondria are important organelles for ROS generation, mitochondrial membrane potential change was then investigated using a Mito-Tracker Red CMXRos. The significant reduction in red fluorescence in the USIB + laser group indicates alterations in mitochondrial membrane potential, revealing mitochondrial dysfunction (Figure 3H). The JC-1 fluorescence probe was employed to further investigate changes in mitochondrial membrane potential. Mitochondria with a healthy, high membrane potential form JC-1 aggregates (red fluorescence), while mitochondria with impaired function have a lower potential and exist as JC-1 monomers (green fluorescence). It is suggested that compared with USIB nanoparticles, that can induce weak changes in mitochondrial membrane potential, and laser irradiation alone having a negligible effect, the combination of USIB + laser causes significant changes (Figure 3I). Changes in mitochondrial membrane potential are an early characteristic of apoptosis, and thus, flow cytometry was finally used, which suggested the apoptotic mode of cell death (Figure 3J).

### 3.6. Biosafety Evaluation

**Figure 4:**
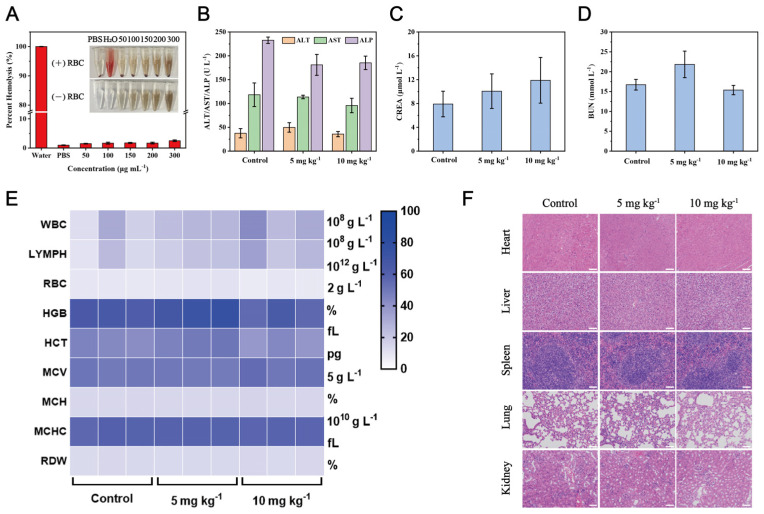
Biosafety evaluation. (**A**) Hemolysis assay with different concentrations of USIB nanoparticles, (*n* = 3). (**B**) Liver function indices, (*n* = 3). Renal function indices CERA (**C**) and BUN (**D**), (*n* = 3). (**E**) Hematological parameters. (**F**) H&E staining of major organs (Scale bar: 50 µm). Data presented as mean ± S.D.

Inspired by the therapeutic effect of USIB nanoparticles in vitro, we proceeded to investigate their safety and effectiveness in vivo. Initially, we assessed the blood compatibility of USIB nanoparticles through hemolysis assays. The results indicated that no hemolysis occurred at the tested concentrations, highlighting the nanoparticles’ excellent compatibility with blood (Figure 4A). To further evaluate their biotoxicity in vivo, we examined blood biochemical markers and hematological parameters. A comprehensive analysis of blood biochemistry and histological assessments revealed that all parameters remained within normal ranges in mice treated with USIB nanoparticles (5 mg kg^−1^, 10 mg kg^−1^) compared to the control group (Figure 4B–E). Additionally, histological examination of major organs, including the heart, liver, spleen, lungs, and kidneys, using H&E staining revealed no significant changes (Figure 4F). These findings confirm the excellent biocompatibility of USIB nanoparticles, ensuring their suitability for in vivo therapeutic applications.

### 3.7. Tumor Therapeutic Effect In Vivo

**Figure 5:**
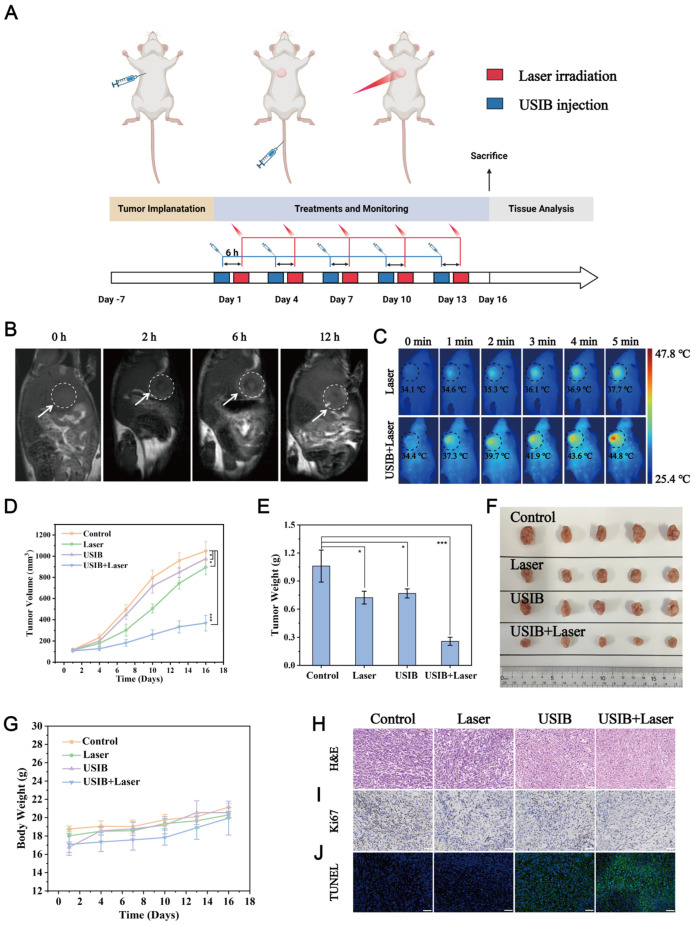
Therapeutic effect of USIB nanoparticles in vivo. (**A**) Schematic diagram of the animal experimental process (this figure was created using BioRender). (**B**) MRI after tail vein injection of USIB nanoparticles (0, 2, 6, 12 h, Tumor positions are indicated by arrows and dashed circle). (**C**) Infrared thermal imaging at different times. (**D**) Tumor growth curves with different treatments (*n* = 5). Tumor weight changes (**E**) and corresponding photograph (**F**) after different treatments (*n* = 5). (**G**) The body weight changes in mice during the period of treatment (*n* = 5). H&E staining (**H**), Ki67 staining (**I**), and TUNEL staining (**J**) of tumor tissue (Scale bar: 50 µm). Data presented as mean ± S.D. Statistical significance was calculated by one-way ANOVA with Tukey’s multiple comparisons test: * *p* < 0.05, *** *p* < 0.001.

Leveraging the favorable biocompatibility of USIB nanoparticles, we evaluated their antitumor effects in a BALB/c mouse model with implanted 4T1 tumors. Mice were randomly assigned to four groups: (Ⅰ) Control, (Ⅱ) Laser, (Ⅲ) USIB, and (Ⅳ) USIB + Laser. Considering the magnetic resonance performance of USIB nanoparticles, T_1_-weighted MR images were acquired before and after tail vein injection. Initially, we investigated the accumulation of USIB nanoparticles in tumor tissue using MRI. Within 6 h post-injection, tumor tissues brightened on T_1_-weighted MR images (23.6% increase compared to pre-injection), indicating enhanced T_1_ relaxation in the tumor (Figure 5B, Figure S12 and S13). This brightening effect persisted for at least 24 h.

The 4T1 tumor-bearing mouse model and drug administration schedule are shown in Figure 5A, with laser irradiation (0.8 W cm^−2^) applied 6 h after intravenous injection (10 mg kg^−1^). The tumor site temperature gradually rose to 44.8 °C after 5 min of irradiation, compared to 37.7 °C in the control group, demonstrating the photothermal conversion capability of USIB nanoparticles in vivo (Figure 5C and Figure S14). The tumor volume curve during treatment showed that the combination of USIB nanoparticles and laser irradiation effectively suppressed tumor growth compared to other groups (Figure 5D and Figure S15). Furthermore, tumor weight and corresponding photographs after treatment aligned with the tumor volume results (Figure 5E and Figure 5F). Consequently, the strategy combining the cascade SOD-POD enzyme-mimic activity of USIB nanoparticles with mild photothermal therapy achieved satisfactory tumor suppression. It is noteworthy that either the laser irradiation or the USIB nanoparticles alone can achieve limited tumor growth inhibition, with 15.71% and 11.26% tumor volume inhibition rates, respective. USIB + laser treatment demonstrates a substantial inhibition of tumor growth, with 64.49% tumor volume inhibition rate. It further evidenced that the combination of CDT and PTT is not just additive, and some synergistic effect presented due to the influence of photothermal effect to enzymatic activity. We attribute this synergy to two mechanisms: (1) mild photothermal stimulation generates localized heat insufficient for standalone tumor ablation but improves tumor microenvironment permeability; (2) laser-triggered activation of electron transfer pathways within USIB nanoparticles amplifies the SOD-POD cascade activity, thereby enhancing ·OH generation and improving CDT efficiency. Additionally, all groups exhibited minimal body weight changes during treatment, indicating minimal adverse effects (Figure 5G). To further validate the therapeutic efficacy of USIB nanoparticles, hematoxylin and eosin (H&E) staining, Ki67 immunostaining, and terminal deoxynucleotidyl transferase-mediated dUTP nick-end labeling (TUNEL) staining were performed (Figure 5H–J). Overall, these results confirm that the combination of USIB nanoparticles and laser irradiation effectively killed tumor tissue, highlighting the potential use of USIB nanoparticles in tumor therapy.

## 4. Conclusions

In summary, USIB nanoparticles were synthesized using a mesoporous silica-mediated hard template method. These nanoparticles demonstrate outstanding SOD and POD enzyme-mimic activity, as well as photothermal conversion capabilities. They increase intracellular ROS levels through a SOD-POD cascade reaction, effectively killing tumor cells. Interestingly, we found that the tumor cell-killing effect of USIB nanoparticles can be further enhanced under laser irradiation. It is important to note that this enhancement is not solely due to the heat generated by the laser. In vitro experiments revealed that the combination of USIB nanoparticles and laser irradiation induces apoptosis by affecting mitochondrial function. Additionally, biosafety experiments have shown that USIB nanoparticles possess excellent biocompatibility. In vivo results demonstrate that USIB nanoparticles significantly suppress tumor growth in mice bearing 4T1 tumors. Overall, this study presents a novel strategy that combines self-cascading enzyme-mimic catalytic reactions with mild photothermal therapy to effectively kill tumor tissue while minimizing damage to normal cells, offering potential new approaches for future cancer treatment.

## Data Availability

The data presented in this paper can be made available upon request to the corresponding author.

## Supplementary Materials

The following supporting information can be downloaded at: https://www.mdpi.com/article/10.3390/ma18071649/s1, Figure S1: Pore size distribution of MSN absorption curve (A) and desorption curve (B); Figure S2: Pore size distribution of MSN@Fe absorption curve (A) and desorption curve (B). (C) TEM image of MSN@Fe; Figure S3: (A) XPS survey and (B) high-resolution N 1s XPS spectrum of USIB nanoparticles; Figure S4: Stability of USIB nanoparticles in aqueous solutions over 7 days. (*n* = 3, mean ± S.D.); Figure S5: UV-Vis absorbance spectrum of USIB nanoparticles at different concentrations; Figure S6: Photothermal conversion ability of USIB nanoparticles (100 μg mL^−1^) under 808 nm laser irradiation; Figure S7: (A) Infrared thermal imaging of USIB nanoparticles under 808 nm laser irradiation (1 W cm^−2^, 10 min) with different concentrations (40, 100, 200, 400, 800 μg mL^−1^). (B) Infrared thermal imaging of USIB nanoparticles (100 μg mL^−1^) under 808 nm laser irradiation (0.5, 1, 1.5 W cm^−2^, 10 min); Figure S8: O_2_·-inhibitor rate of O_2_—of USIB nanoparticles measured by pyrogallol assay (*n* = 3, mean ± S.D.); Figure S9: Standard curve of H_2_O_2_ concentration. (*n* = 3, mean ± S.D.); Figure S10: Degradation curves of MB treated with USIB nanoparticles; Figure S11: (A) Magnetization curves (M-H) of USIB nanoparticles. (B) Plot of 1/T2 over Fe concentration of USIB nanoparticles; Figure S12: MRI after tail vein injection of USIB nanoparticles (1, 4, 8, 24 h); Figure S13: Intensity changes of T1-weighted MR signal after injection of USIB nanoparticles (*n* = 3, mean ± S.D.); Figure S14: Photothermal heating curves under different treatments; Figure S15: Tumor growth curves with different treatments.

## Abbreviations

The following abbreviations are used in this manuscript:

CDT	Chemodynamic therapy
H_2_O_2_	Hydrogen peroxide
USIB	Ultra-small iron-based
SOD	Superoxide dismutase
POD	Peroxidase
O_2_^·−^	Superoxide anions
·OH	Hydroxyl radicals
TME	Tumor microenvironment
CAT	Catalase
GPx	Glutathione peroxidase
PTT	Photothermal therapy
TEOS	Tetraethyl orthosilicate
CTAB	Hexadecyl trimethyl ammonium bromide
TEA	Trolamine
NaOH	Sodium hydroxide
MB	Methylene blue
TMB	3,3′,5,5′-tetramethylbenzidine
NAC	N-acetyl cysteine
DMSO	Dimethyl sulfoxide
MTT	Methylthiazolyldiphenyl-tetrazolium bromide
MSN	Mesoporous silica nanoparticle
TEM	Transmission electron microscopy
XRD	X-ray diffraction
XPS	X-ray photoelectron spectroscopy
BET	Brunauer–Emmett–Teller
FT-IR	Fourier transform infrared
UV-Vis	Ultraviolet-visible
NIR	Near-infrared
ITI	Infrared thermal imaging
4T1	Mouse breast cancer
L929	Mouse fibroblast
ROS	Reactive oxygen species
MRI	Magnetic Resonance Image
H&E	Hematoxylin and eosin
TUNEL	Terminal deoxynucleotidyl transferase-mediated dUTP nick-end labeling

## References

[1] Zhou Y., Fan S., Feng L., Huang X., Chen X. (2021). Manipulating Intratumoral Fenton Chemistry for Enhanced Chemodynamic and Chemodynamic-Synergized Multimodal Therapy. Adv. Mater..

[2] Tang Z., Liu Y., He M., Bu W. (2019). Chemodynamic Therapy: Tumour Microenvironment-Mediated Fenton and Fenton-like Reactions. Angew. Chem. Int. Ed..

[3] Li S.L., Jiang P., Jiang F.L., Liu Y. (2021). Recent Advances in Nanomaterial-Based Nanoplatforms for Chemodynamic Cancer Therapy. Adv. Funct. Mater..

[4] Chen W., Yang M., Li J., Chen Z., Hu L., Zhang J., Cai L., Qiu L., Chen J. (2023). GSH-Activatable Metal-Phenolic Networks for Photothermal-Enhanced Chemotherapy and Chemodynamic Therapy. J. Funct. Biomater..

[5] Yang G., Xu L., Chao Y., Xu J., Sun X., Wu Y., Peng R., Liu Z. (2017). Hollow MnO_2_ as a tumor-microenvironment-responsive biodegradable nano-platform for combination therapy favoring antitumor immune responses. Nat. Commun..

[6] Huo M., Wang L., Chen Y., Shi J. (2017). Tumor-selective catalytic nanomedicine by nanocatalyst delivery. Nat. Commun..

[7] Di X., Pei Z., Pei Y., James T.D. (2023). Tumor microenvironment-oriented MOFs for chemodynamic therapy. Coord. Chem. Rev..

[8] Fan H., Guo Z. (2023). Tumor microenvironment-responsive manganese-based nanomaterials for cancer treatment. Coord. Chem. Rev..

[9] Yang B., Chen Y., Shi J. (2019). Nanocatalytic Medicine. Adv. Mater..

[10] Wang X., Zhong X., Liu Z., Cheng L. (2020). Recent progress of chemodynamic therapy-induced combination cancer therapy. Nano Today.

[11] Gao H., Cao Z., Liu H., Chen L., Bai Y., Wu Q., Yu X., Wei W., Wang M. (2023). Multifunctional nanomedicines-enabled chemodynamic-synergized multimodal tumor therapy via Fenton and Fenton-like reactions. Theranostics.

[12] Qi J., Jiang G., Wan Y., Liu J., Pi F. (2023). Nanomaterials-modulated Fenton reactions: Strategies, chemodynamic therapy and future trends. Chem. Eng. J..

[13] Liu J., Dong S., Gai S., Dong Y., Liu B., Zhao Z., Xie Y., Feng L., Yang P., Lin J. (2023). Design and Mechanism Insight of Monodispersed AuCuPt Alloy Nanozyme with Antitumor Activity. ACS Nano.

[14] Yan R., Ren J., Wen J., Cao Z., Wu D., Qin M., Xu D., Castillo R., Li F., Wang F. (2022). Enzyme Therapeutic for Ischemia and Reperfusion Injury in Organ Transplantation. Adv. Mater..

[15] Wu S., Nan F., Zhang K., Hao W., Shi D., Li Y., Deng W., Jarhen N., Li K., Xiao Y. (2024). A novel metal-organic framework encapsulated iridium oxide nanozyme enhanced antisense oligonucleotide combo for osteoarthritis synergistic therapy. Aggregate.

[16] Wang S., Cheng M., Wang S., Jiang W., Yang F., Shen X., Zhang L., Yan X., Jiang B., Fan K. (2024). A Self-Catalytic NO/O_2_ Gas-Releasing Nanozyme for Radiotherapy Sensitization through Vascular Normalization and Hypoxia Relief. Adv. Mater..

[17] Yu B., Wang W., Sun W., Jiang C., Lu L. (2021). Defect Engineering Enables Synergistic Action of Enzyme-Mimicking Active Centers for High-Efficiency Tumor Therapy. J. Am. Chem. Soc..

[18] Yang H., Lin P., Zhang B., Li F., Ling D. (2024). A Nucleophilicity-Engineered DNA Ligation Blockade Nanoradiosensitizer Induces Irreversible DNA Damage to Overcome Cancer Radioresistance. Adv. Mater..

[19] Jiang P., Zhang L., Liu X., Ye C., Zhu P., Tan T., Wang D., Wang Y. (2024). Tuning oxidant and antioxidant activities of ceria by anchoring copper single-site for antibacterial application. Nat. Commun..

[20] Li Z., Ding B., Li J., Chen H., Zhang J., Tan J., Ma X., Han D., Ma P., Lin J. (2024). Multi-Enzyme Mimetic MoCu Dual-Atom Nanozyme Triggering Oxidative Stress Cascade Amplification for High-Efficiency Synergistic Cancer Therapy. Angew. Chem. Int. Ed..

[21] Demkiv O., Nogala W., Stasyuk N., Grynchyshyn N., Vus B., Gonchar M. (2023). The Peroxidase-like Nanocomposites as Hydrogen Peroxide-Sensitive Elements in Cholesterol Oxidase-Based Biosensors for Cholesterol Assay. J. Funct. Biomater..

[22] Li X., Ding B., Li J., Han D., Chen H., Tan J., Meng Q., Zheng P., Ma P.A., Lin J. (2024). Valence-tailored copper-based nanoparticles for enhanced chemodynamic therapy through prolonged ROS generation and potentiated GSH depletion. Nano Res..

[23] Zhou J., Xu D., Tian G., He Q., Zhang X., Liao J., Mei L., Chen L., Gao L., Zhao L. (2023). Coordination-Driven Self-Assembly Strategy-Activated Cu Single-Atom Nanozymes for Catalytic Tumor-Specific Therapy. J. Am. Chem. Soc..

[24] Sang Y., Cao F., Li W., Zhang L., You Y., Deng Q., Dong K., Ren J., Qu X. (2020). Bioinspired Construction of a Nanozyme-Based H_2_O_2_ Homeostasis Disruptor for Intensive Chemodynamic Therapy. J. Am. Chem. Soc..

[25] Shen H., Fu Y., Liu F., Zhang W., Yuan Y., Yang G., Yang M., Li L. (2024). AuCePt porous hollow cascade nanozymes targeted delivery of disulfiram for alleviating hepatic insulin resistance. J. Nanobiotechnol..

[26] Liu Y., Liu Y., Shi P., Hu X., Fan X., Wu Y., Pan J., Bai Q., Li Q. (2024). Single-atom nanozyme liposome-integrated microneedles for in situ drug delivery and anti-inflammatory therapy in Parkinson’s disease. J. Nanobiotechnol..

[27] Gao F., Liang W., Chen Q., Chen B., Liu Y., Liu Z., Xu X., Zhu R., Cheng L. (2024). A Curcumin-Decorated Nanozyme with ROS Scavenging and Anti-Inflammatory Properties for Neuroprotection. Nanomaterials.

[28] Liu H., Zhang Y., Zhang M., Yu Z., Zhang M. (2023). Oral Administration of Platinum Nanoparticles with SOD/CAT Cascade Catalytic Activity to Alleviate Ulcerative Colitis. J. Funct. Biomater..

[29] Sheng J., Wu Y., Ding H., Feng K., Shen Y., Zhang Y., Gu N. (2024). Multienzyme-Like Nanozymes: Regulation, Rational Design, and Application. Adv. Mater..

[30] Liu J., Chen Z., Liu H., Qin S., Li M., Shi L., Zhou C., Liao T., Li C., Lv Q. (2024). Nickel-Based Metal-Organic Frameworks Promote Diabetic Wound Healing via Scavenging Reactive Oxygen Species and Enhancing Angiogenesis. Small.

[31] Shi T., Cui Y., Yuan H., Qi R., Yu Y. (2023). Burgeoning Single-Atom Nanozymes for Efficient Bacterial Elimination. Nanomaterials.

[32] Xu Y., An X., Liu L., Cao X., Wu Z., Jia W., Sun J., Wang H., Huo J., Sun Z. (2024). Self-Cascade Redox Modulator Trilogically Renovates Intestinal Microenvironment for Mitigating Endotoxemia. ACS Nano.

[33] Wang W., Zhang L., Zhang Y., Liu X., Song A., Ren J., Qu X. (2024). A Self-Adaptive Pyroptosis Inducer: Optimizing the Catalytic Microenvironment of Nanozymes by Membrane-Adhered Microbe Enables Potent Cancer Immunotherapy. Adv. Mater..

[34] Kim C.K., Kim T., Choi I., Soh M., Kim D., Kim Y., Jang H., Yang H., Kim J.Y., Park H. (2012). Ceria Nanoparticles that can Protect against Ischemic Stroke. Angew. Chem. Int. Ed..

[35] Liu Z., Xie L., Qiu K., Liao X., Rees T.W., Zhao Z., Ji L., Chao H. (2020). An Ultrasmall RuO_2_ Nanozyme Exhibiting Multienzyme-like Activity for the Prevention of Acute Kidney Injury. ACS Appl. Mater. Inter..

[36] Liu T., Xiao B., Xiang F., Tan J., Chen Z., Zhang X., Wu C., Mao Z., Luo G., Chen X. (2020). Ultrasmall copper-based nanoparticles for reactive oxygen species scavenging and alleviation of inflammation related diseases. Nat. Commun..

[37] Cui X., Ruan Q., Zhuo X., Xia X., Hu J., Fu R., Li Y., Wang J., Xu H. (2023). Photothermal Nanomaterials: A Powerful Light-to-Heat Converter. Chem. Rev..

[38] Fan W., Yung B., Huang P., Chen X. (2017). Nanotechnology for Multimodal Synergistic Cancer Therapy. Chem. Rev..

[39] Jana D., He B., Chen Y., Liu J., Zhao Y. (2022). A Defect-Engineered Nanozyme for Targeted NIR-II Photothermal Immunotherapy of Cancer. Adv. Mater..

[40] Xue C., Li M., Liu C., Li Y., Fei Y., Hu Y., Cai K., Zhao Y., Luo Z. (2021). NIR-Actuated Remote Activation of Ferroptosis in Target Tumor Cells through a Photothermally Responsive Iron-Chelated Biopolymer Nanoplatform. Angew. Chem. Int. Ed..

[41] Niu X., Zhu Y., Ding C., Ma J., Wei P., Lin Y., Fang W., He Q., Li C., Cheng J. (2023). Dual-Active Center AgFeCu Nanocatalyst for Tumor Destruction via Self-Catalytically Enhanced Mild Photothermal Therapy. Adv. Funct. Mater..

[42] Dong S., Dong Y., Zhao Z., Liu J., Liu S., Feng L., He F., Gai S., Xie Y., Yang P. (2023). “Electron Transport Chain Interference” Strategy of Amplified Mild-Photothermal Therapy and Defect-Engineered Multi-Enzymatic Activities for Synergistic Tumor-Personalized Suppression. J. Am. Chem. Soc..

[43] Tao N., Jiao L., Li H., Deng L., Wang W., Zhao S., Chen W., Chen L., Zhu C., Liu Y. (2023). A Mild Hyperthermia Hollow Carbon Nanozyme as Pyroptosis Inducer for Boosted Antitumor Immunity. ACS Nano.

[44] (2019). Nanotechnologies-Measurementmethod for Peroxidase-like Activity of Iron Oxide Nanoparticles.

[45] Yang L., Tian B., Xie Y., Dong S., Yang M., Gai S., Lin J. (2023). Oxygen-Vacancy-Rich Piezoelectric BiO_2−_*x* Nanosheets for Augmented Piezocatalytic, Sonothermal, and Enzymatic Therapies. Adv. Mater..

[46] Shi X., Liu Q., Gong Z., Jin Z., Guo H., Wen X., Li C. (2024). Vacancy-rich Fe-Ce mixed oxides as nanoenzymes for efficient antioxidant activity in vitro. Appl. Surf. Sci..

[47] Hu L., Sun W., Tang Y., Li S., Zhang B., Sun X., Ji W., Ma L., Deng H., Han S. (2021). Photothermal effect enhancing graphene quantum dots/semiconducting polymer/nanozyme-mediated cancer catalytic therapy. Carbon.

[48] Vyas A., Petrášek Z., Nidetzky B. (2024). Limits of Non-invasive Enzymatic Activation by Local Temperature Control. Small.

[49] Chen Q., He S., Zhang F., Cui F., Liu J., Wang M., Wang D., Jin Z., Li C. (2021). A versatile Pt-Ce6 nanoplatform as catalase nanozyme and NIR-II photothermal agent for enhanced PDT/PTT tumor therapy. Sci. China Mater..

[50] Pan M.M., Li P., Yu Y.P., Jiang M., Yang X., Zhang P., Nie J., Hu J., Yu X., Xu L. (2023). Bimetallic Ions Functionalized Metal–Organic-Framework Nanozyme for Tumor Microenvironment Regulating and Enhanced Photodynamic Therapy for Hypoxic Tumor. Adv. Healthc. Mater..

[51] Dai R., Liu Q., Zhang B., Zhang X., Gao M., Li D., Kang W., Chen L., Zhao M., Zheng Z. (2024). A Single NIR-II Laser-Triggered Self-Enhancing Photo/Enzyme-coupled Three-in-One Nanosystems for Breast Cancer Phototheranostics. Adv. Healthc. Mater..

[52] Kim B.H., Lee N., Kim H., An K., Park Y.I., Choi Y., Shin K., Lee Y., Kwon S.G., Na H.B. (2011). Large-Scale Synthesis of Uniform and Extremely Small-Sized Iron Oxide Nanoparticles for High-Resolution *T*_1_ Magnetic Resonance Imaging Contrast Agents. J. Am. Chem. Soc..

[53] Choi J.S., Kim S., Yoo D., Shin T.H., Kim H., Gomes M.D., Kim S.H., Pines A., Cheon J. (2017). Distance-dependent magnetic resonance tuning as a versatile MRI sensing platform for biological targets. Nat. Mater..

[54] Yu J., Zhao F., Gao W., Yang X., Ju Y., Zhao L., Guo W., Xie J., Liang X., Tao X. (2019). Magnetic Reactive Oxygen Species Nanoreactor for Switchable Magnetic Resonance Imaging Guided Cancer Therapy Based on pH-Sensitive Fe_5_C_2_@Fe_3_O_4_ Nanoparticles. ACS Nano.

