# Supplementary material for: Ultra-Small Iron-Based Nanoparticles with Mild Photothermal-Enhanced Cascade Enzyme-Mimic Reactions for Tumor Therapy
Source: Materials (Basel). 2025 Apr 3;18(7):1649. doi: 10.3390/ma18071649 (PMC11990581; doi:10.3390/ma18071649)
Supplement: Supplementary file 1 [file materials-18-01649-s001.zip › materials-3529084-supplementary.pdf]

# Supporting Information

## Ultra-Small Iron-Based Nanoparticles with Mild Photothermal Enhanced Cascade Enzyme-Mimic Reactions for Tumor Therapy

Jing Yu [1,2*,#], Shuangshan Li [1,2#], Xun Zhu [1,2], Hongyan Yu [1,2], Hao Gao [1,2], Jiarui Qi [1,2], Yao Ying [1,2], Liang Qiao [1,2], Jingwu Zheng [1,2], Juan Li [1,2], Jing Yu [1,2*], Shenglei Che [1,2*]

#These authors contributed equally to this work

[1] Research Center of Magnetic and Electronic Materials, Zhejiang University of Technology, Hangzhou, 310014, China

[2] College of Materials Science and Engineering, Zhejiang University of Technology, Hangzhou, 310014, China

*Corresponding author:

Jing Yu

yujing@zjut.edu.cn

Shenglei Che

cheshenglei@zjut.edu.cn

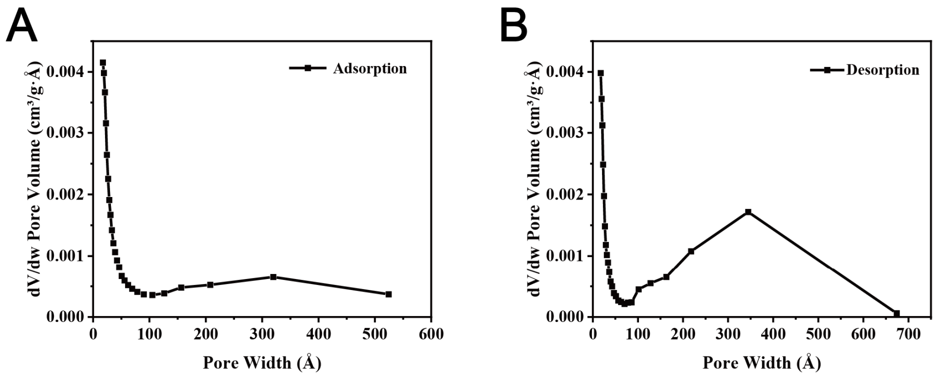


**Figure S1.** Pore size distribution of MSN absorption curve (A) and desorption curve (B).

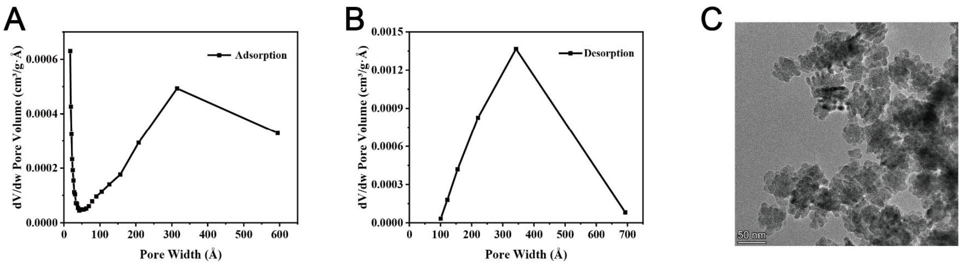


**Figure S2.** Pore size distribution of MSN@Fe absorption curve (A) and desorption curve (B). (C) TEM image of MSN@Fe.

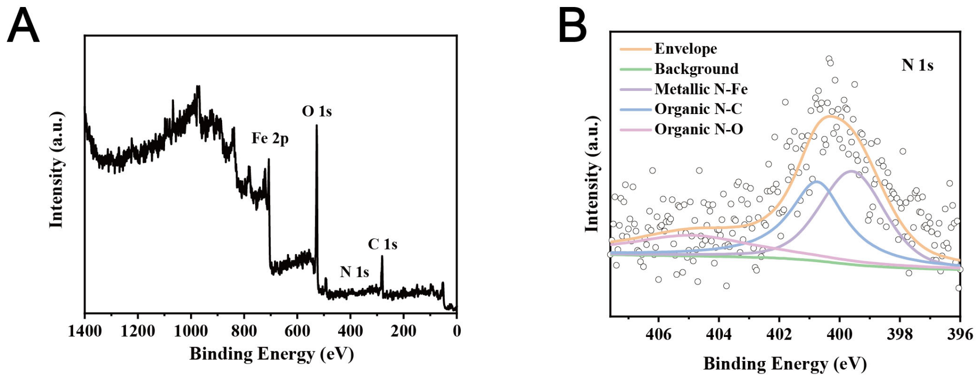


**Figure S3.** (A) XPS survey and (B) high-resolution N 1s XPS spectrum of USIB nanoparticles.

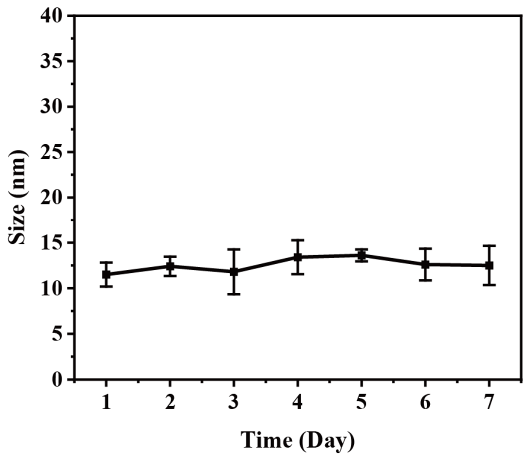


**Figure S4.** Stability of USIB nanoparticles in aqueous solutions over 7 days. ($n$ = 3, mean ± S.D.).

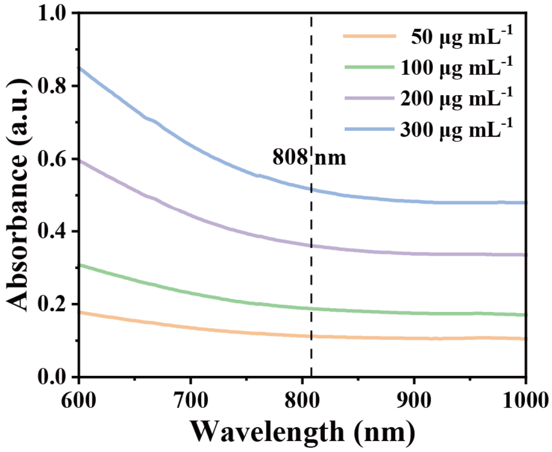


**Figure S5.** UV-Vis absorbance spectrum of USIB nanoparticles at different concentrations.

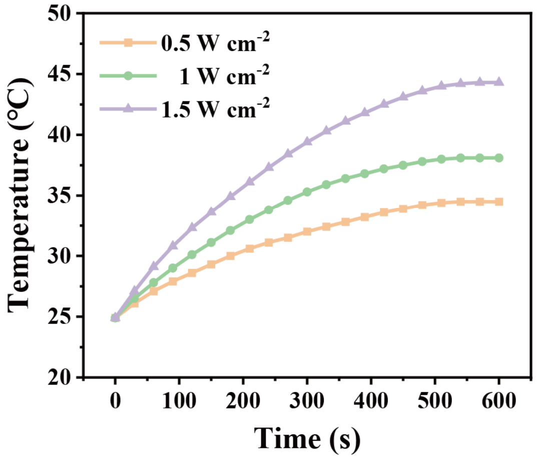


**Figure S6.** Photothermal conversion ability of USIB nanoparticles (100 μg $mL^{-1}$) under 808 nm laser irradiation.

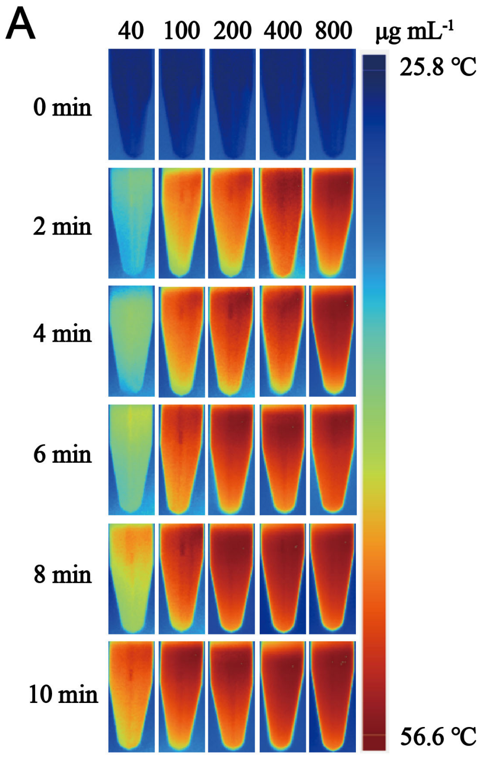


**Figure S7.** (A) Infrared thermal imaging of USIB nanoparticles under 808 nm laser irradiation (1 W cm$^{-2}$, 10 min) with different concentrations (40, 100, 200, 400, 800 μg mL$^{-1}$). (B) Infrared thermal imaging of USIB nanoparticles (100 μg mL$^{-1}$) under 808 nm laser irradiation (0.5, 1, 1.5 W cm$^{-2}$, 10 min).

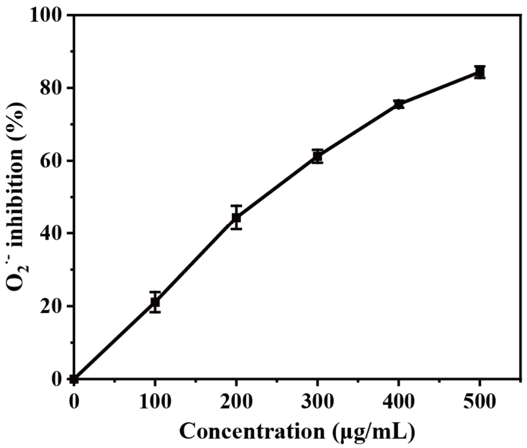


**Figure S8.** $O_2^{\cdot-}$inhibitor rate of $O_2^{\cdot-}$ of USIB nanoparticles measured by pyrogallol assay (*n* = 3, mean ± S.D.).

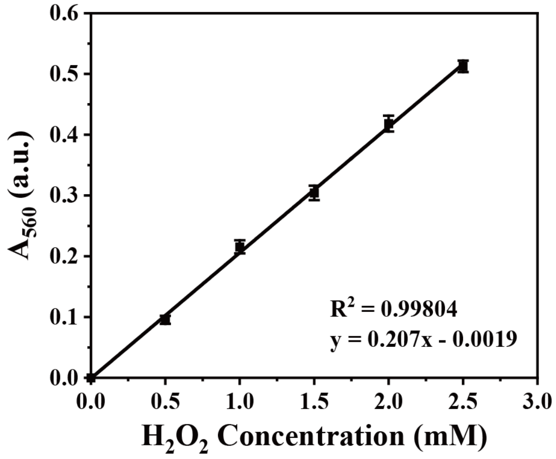


**Figure S9.** Standard curve of $H_2O_2$ concentration. (*n* = 3, mean ± S.D.).

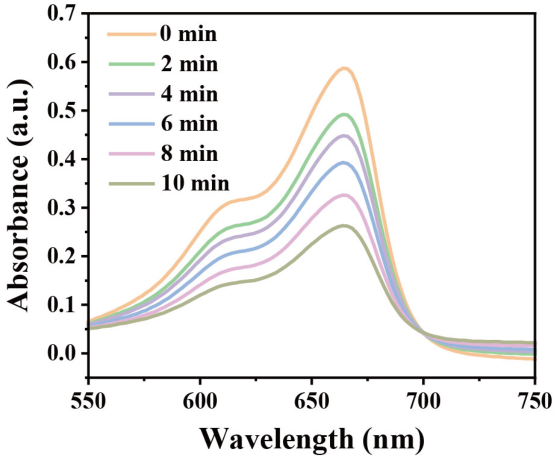


**Figure S10.** Degradation curves of MB treated with USIB nanoparticles.

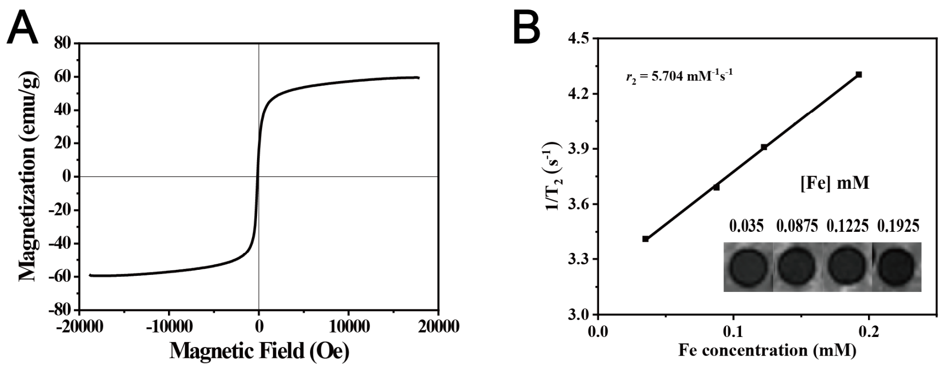


**Figure S11.** (A) Magnetization curves (M-H) of USIB nanoparticles. (B) Plot of $1/T_2$ over Fe concentration of USIB nanoparticles.

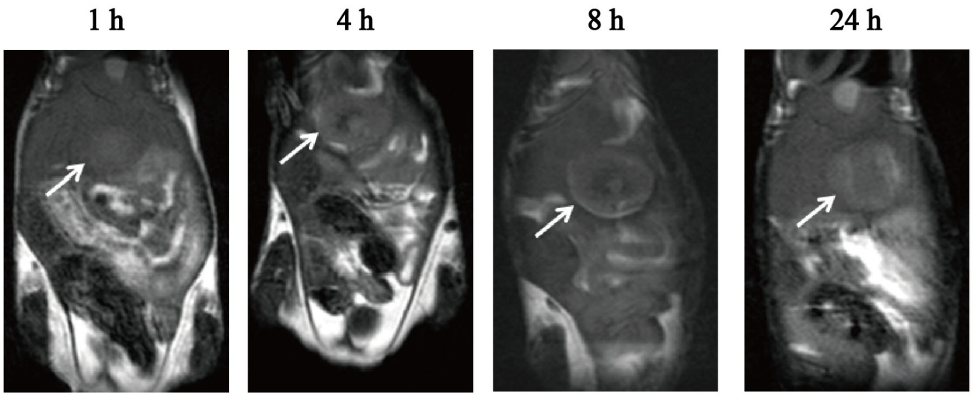


**Figure S12.** MRI after tail vein injection of USIB nanoparticles (1, 4 , 8, 24 h).

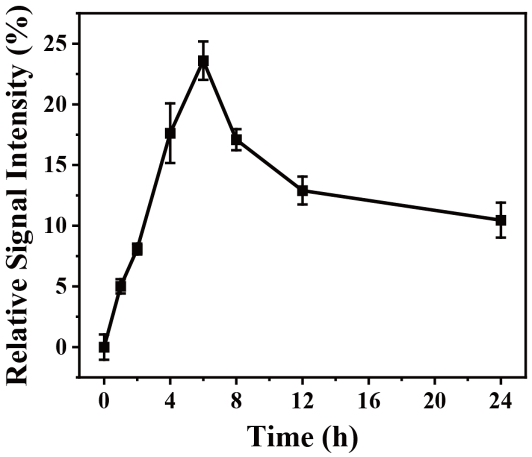


**Figure S13.** Intensity changes of $T_1$-weighted MR signal after injection of USIB nanoparticles ($n$ = 3, mean ± S.D.).

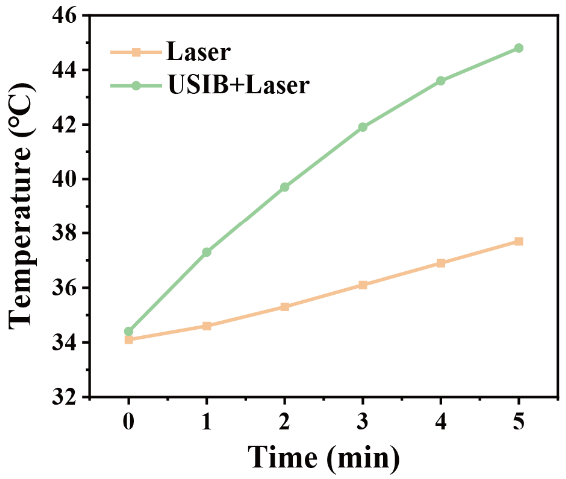


**Figure S14.** Photothermal heating curves under different treatments.

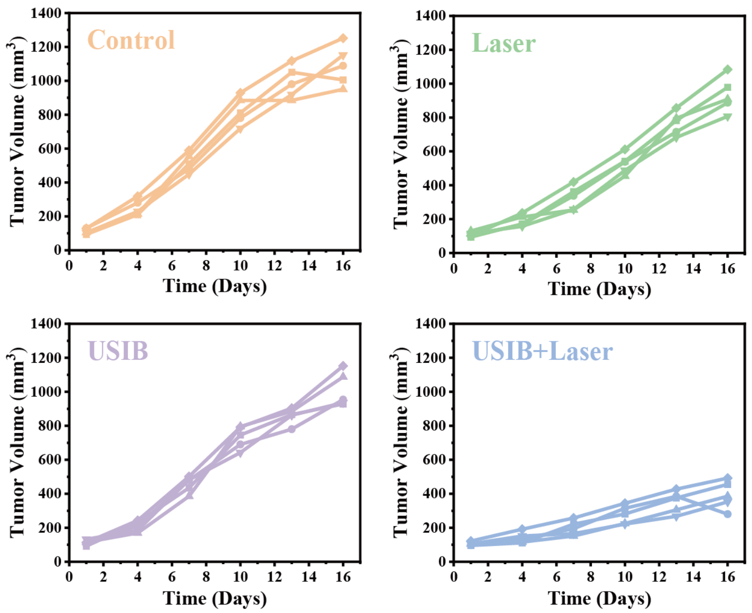


**Figure S15.** Tumor growth curves with different treatments.
